# Transylvania Medical Journal

**Published:** 1852-12

**Authors:** 


*Transylvania Medical Journal.* — The editorial management of this periodical has lately been transferred to Dr. L. J. Frazee, formerly of Maysville, Ky. It has been enlarged and improved in appearance. Its cotemporary in the same city (Louisville) states that Dr. Frazee is "a pleasant writer, a gentleman of attainments, ambitious of eminence in his profession." He is author of a small volume, published, a few years ago, on his return from Europe, entitled "The Medical Student in Paris."

